# Marine amphipods as a new live prey for ornamental aquaculture: exploring the potential of *Parhyale hawaiensis* and *Elasmopus pectenicrus*

**DOI:** 10.7717/peerj.10840

**Published:** 2021-02-10

**Authors:** Jorge Arturo Vargas-Abúndez, Humberto Ivan López-Vázquez, Maite Mascaró, Gemma Leticia Martínez-Moreno, Nuno Simões

**Affiliations:** 1 Posgrado en Ciencias del Mar y Limnología, Universidad Nacional Autónoma de México, Mexico City, Ciudad de Mexico, Mexico; 2 Unidad Multidisciplinaria de Docencia e Investigacion de Sisal, Facultad de Ciencias, Universidad Nacional Autónoma de México, Sisal, Yucatán, Mexico; 3 Laboratorio de Resiliencia Costera (LANRESC, CONACYT), Universidad Nacional Autónoma de México, Sisal, Yucatán, Mexico; 4 International Chair for Coastal and Marine Studies in Mexico, Harte Research Institute for Gulf of Mexico Studies, Texas A&M University-Corpus Christi, Corpus Christi, TX, United States of America

**Keywords:** Gammarids, Reproductive biology, Sexual maturity, Sex ratio, Fecundity, Energy content, Cannibalism, Gulf of Mexico, Mexico, Yucatán

## Abstract

Marine amphipods are gaining attention in aquaculture as a natural live food alternative to traditional preys such as brine shrimps (*Artemia* spp.). The use of *Artemia* is convenient for the culture of many marine species, but often problematic for some others, such as seahorses and other marine ornamental species. Unlike *Artemia*, marine amphipods are consumed by fish in their natural environment and show biochemical profiles that better match the nutritional requirements of marine fish, particularly of polyunsaturated fatty acids (PUFA), including eicosapentaenoic (EPA) and docosahexaenoic (DHA) acids. Despite their potentially easy culture, there are no established culture techniques and a deeper knowledge on the reproductive biology, nutritional profiles and culture methodologies is still needed to potentiate the optimization of mass production. The present study assessed, for the first time, the aquaculture potential of *Parhyale hawaiensis* and *Elasmopus pectenicrus*, two cosmopolitan marine gammarids (as per traditional schemes of classification) that naturally proliferate in the wild and in aquaculture facilities. For that purpose, aspects of the population and reproductive biology of the species were characterized and then a series of laboratory-scale experiments were conducted to determine amphipod productivity, the time needed to reach sexual maturity by hatchlings (generation time), cannibalism degree, the effects of sex ratio on fecundity and the effects of diet (shrimp diet, plant-based diet and commercial fish diet) on fecundity and juvenile growth. *P. hawaiensis*, unlike *E. pectenicrus*, was easily maintained and propagated in laboratory conditions. *P. hawaiensis* showed a higher total length (9.3 ± 1.3 mm), wet weight (14.4 ± 6.2 mg), dry weight (10.5 ± 4.4 mg), females/males sex ratio (2.24), fecundity (12.8 ± 5.7 embryos per female), and gross energy content (16.71 ± 0.67 kJ g-1) compared to *E. pectenicrus* (7.9 ± 1.2 mm total length; 8.4 ± 4.3 mg wet weight; 5.7 ± 3.2 mg dry weight; 1.34 females/males sex ratio; 6.5 ± 3.9 embryos per female; 12.86 ± 0.82 kJ g^−1^ gross energy content). *P. hawaiensis* juvenile growth showed a small, but significant, reduction by the use of a plant-based diet compared to a commercial shrimp and fish diet; however, fecundity was not affected, supporting the possible use of inexpensive diets to mass produce amphipods as live or frozen food. Possible limitations of *P. hawaiensis* could be their quite long generation times (50.9 ± 5.8 days) and relatively low fecundity levels (12.8 ± 5.7 embryos per female). With an observed productivity rate of 0.36 ± 0.08 juveniles per amphipod couple per day, *P. hawaiensis* could become a specialty feed for species that cannot easily transition to a formulated diet such as seahorses and other highly priced marine ornamental species.

## Introduction

Live food organisms are commonly used for the early rearing of fish and invertebrates, especially the larval stages (Southgate, 2019). Most larvae are visual feeders adapted to attack moving prey and, especially for altricial larvae, their digestive system is still rudimentary with limited digestive capacities (Conceição et al., 2010). As they develop into adults, the need for live feed decreases as they start to successfully accept inert diets or formulated feed (Hill, Pernetta & Crooks, 2020). However, some ornamental species are not easy (or even impossible) to feed with pelleted aquafeeds (Olivotto et al., 2017). Seahorses, for example, do not readily accept pelleted diets due to their highly specialized feeding behavior, and (unpublished) trials that show some success are not sufficiently successful as yet to replace live/frozen foods (Wilson, Carter & Purser, 2006; Koldewey & Martin-Smith, 2010). Similarly, the use of live/frozen foods, mainly *Artemia* and mysid shrimps, is mandatory for the successful early culture of some cephalopod, lobster and shrimp species (Baeza-Rojano et al., 2010; Baeza-Rojano et al., 2013b; Wang & Jeffs, 2014; Planas et al., 2017). Conversely, the successful reproduction of several ornamental species in captivity, such as the Pacific blue tang (*Paracanthurus hepatus)*, is challenging and require the use of high quality live or frozen food organisms included in the broodstock diet (Craig, Gardner & Carnevali, 2017; DiMaggio et al., 2017; Planas et al., 2020).

Several live feeds are available for commercial aquaculture, however, *Artemia* spp. and rotifers are by far the most commonly used, as they can be easily cultured in large quantities at high densities (Planas, Olivotto & Turchi, 2017). Unfortunately, they do not always match the nutritional requirements of all commercially important species during their different stages of development (Conceição et al., 2010). With the current expansion of the aquaculture industry, it is imperative to explore new alternatives both to overcome the lack of suitable foods and to contribute to the diversification of species farmed in aquaculture (Conceição et al., 2010; Southgate, 2019).

Marine amphipods have recently gained attention as alternative live feed in aquaculture, since they are an important natural diet of many marine fish of commercial interest (Amara et al., 2001; Teixeira, Musick & Musik, 2001; Pita, Gamito & Erzini, 2002) and because their culture may potentially be easier and cheaper than commonly used live feeds (Woods, 2009; Harlıoğlu & Farhadi, 2018). This diverse group of benthic crustaceans are mostly detritivores and can tolerate a wide range of environmental factors such as temperature and salinity (Steele & Steele, 1991; Ashton et al., 2007; Cook, Willis & Lozano-Fernandez, 2007). They can be found in high numbers (>100,000 individuals m^2^) in their natural habitats despite their low fecundity (Oliva-Rivera, 2003; Lourido, Moreira & Troncoso, 2008; Woods, 2009; Vázquez-Luis, Sanchez-Jerez & Bayle-Sempere, 2013), and exhibit reproductive strategies such as direct development of the embryo within the marsupium and parental care that may be convenient for culture purposes (Johnson, Stevens & Watling, 2001; Browne et al., 2005). Unlike some used preys, amphipods are naturally rich in lipids, including EPA and DHA (Baeza-Rojano, Hachero-Cruzado & Guerra-García, 2014; Fernandez-Gonzalez et al., 2018; Alberts-Hubatsch, Slater & Beermann, 2019), which are main structural components of cellular membranes and precursors of bioactive molecules, thus playing a pivotal role in fish development and reproduction (Izquierdo, 1996). Several amphipod species have been successfully tested as fishmeal replacement (Moren et al., 2006; Suontama et al., 2007; Harlıoğlu & Farhadi, 2018) and as live/whole feed for seahorses (Murugan et al., 2009; Vargas-Abúndez, Simões & Mascaró, 2018), octopuses (Baeza-Rojano et al., 2013b) and cuttlefish (Baeza-Rojano et al., 2010), revealing promising results.

Most attempts to culture amphipods have been performed under laboratory conditions and with the aim to produce model organisms for toxicological studies (Yuh Lee, 1977; Neuparth, Costa & Costa, 2002; Hyne, Gale & King, 2005; Ashton et al., 2007). Until recently, several studies have explored the culture potential of amphipods for use in aquaculture. These include studies on large-scale culture of caprellids, also known as skeleton shrimps, *Caprella scaura* (Baeza-Rojano et al., 2013a), culture of *C. mutica* in a public aquarium (Nakajima & Takeuchi, 2008), amphipod culture (several species) within an offshore facility applying the integrated multi-trophic aquaculture concept (Guerra-García et al., 2016; Fernandez-Gonzalez et al., 2018), and evaluating the effects of cheap diets and temperature using gammarid species (Xue et al., 2018; Alberts-Hubatsch, Slater & Beermann, 2019). There are no established culture techniques for aquaculture purposes to date and a deeper knowledge on the reproductive biology, nutritional profile and culture methodology of species with aquaculture potential is still needed. This knowledge is essential to scale up the current captive production of amphipods, ensuring efficient production of biomass with an economically feasible output.

*Parhyale hawaiensis* (Dana, 1853) and *Elasmopus pectenicrus* (Bate, 1862) are two gammarids (as per traditional schemes of classification; Martin & David, 2001) with a cosmopolitan distribution in tropical and temperate marine areas (WoRMS Editorial Board, 2020). They are commonly found forming colonies (>7,000 individuals m^2^) in soft- and hard-bottoms as well as in artificial structures with high complexity where they can hide in the presence of detritus and vegetal material such as mangrove leaves (Poovachiranon, Boto & Duke, 1986; Zakhama-Sraieb & Charfi-Cheikhrouha, 2010; Paz-Ríos, Simões & Ardisson, 2013). *P. hawaiensis* is an emerging crustacean model for toxicological, evolutionary and developmental research (Sun & Patel, 2019). Detailed information of the embryonic development is available (Browne et al., 2005), as well as information on laboratory culture of embryos for toxicological tests (Lee & Nicol, 1980). It is presently cultured only at the laboratory-scale (Artal et al., 2018). *E. pectenicrus*, in contrast, has been poorly studied with no prior published information on its reproductive biology or culture methods. However, a recent study suggested that this species could be an interesting candidate as alternative food to live *Artemia*, as feeding behavior of juvenile seahorses (*Hippocampus erectus*) fed this species significantly improved in terms of feeding rate and food intake compared to *Artemia* (Vargas-Abúndez, Simões & Mascaró, 2018).

The aim of the present study was to examine the aquaculture potential of *P. hawaiensis* and *E. pectenicrus* to be used as live fish feeds. These amphipod species were selected because they naturally proliferate at the aquaculture research facilities of the National Autonomous University of Mexico (UNAM) located in Sisal, Yucatán, Mexico, and surrounding coastal areas. Amphipods from an ongoing culture in large out-door tanks have been used as main food item in these facilities for several years with the capacity to sustain superior growth and development compared to enriched *Artemia*. The first section of the present study characterizes biological/reproductive parameters of amphipods occurring in aquaculture facilities, namely body size, weight, sex ratio, fecundity and gross energy content. Afterward, a series of laboratory-scale experiments were performed to know the amphipod age and size at the onset of sexual maturity, amphipod productivity, the possible negative effect of cannibalism on amphipod productivity, the effects of sex ratio on fecundity and the effect of diet on the fecundity and on the juveniles growth. The results provide basic knowledge required to assess the species’ aquaculture potential and are useful to design preliminary strategies for aquaculture pioneer operations. This study contributes to the finding and development of new live foods alternative to traditional ones, such as adult *Artemia*.

## Materials & Methods

### Amphipod collection and maintenance

Amphipods (*P. hawaiensis* and *E. pectenicrus*) were collected from outdoor flow-through systems in which amphipods grow freely, at the fish and octopus aquaculture facilities of the UNAM, located in Sisal, Yucatán, México. They are abundant in green intertidal algae attached to rocks in Sisal beach, from where they likely infiltrate the aquaculture systems. The number of collected amphipods was not recorded, but *P. hawaiensis* dominated in the samples, compared to *E. pectenicrus*. Collected amphipods from the different systems were pooled by species and acclimatized to laboratory conditions. Within the next two days, some amphipods were used for biological/reproductive analyses (i.e., body size, weight, sex ratio, fecundity and gross energy content), while the rest were cultivated to be used as stocking animals for the different experiments performed in the present study. For that purpose, each species was maintained in a round fiberglass tank (79 cm diameter, 35 cm height) with 171 L of filtered (5 µm) seawater. Water in the tanks was maintained at (mean ± standard deviation) 26.0 ± 0.5 °C, salinity 37.2 ± 0.5 ppt, pH 8.1–8.3, NO${}_{2}^{-}$ <0.3 mg L^−1^, NO${}_{3}^{-}$ <5 mg L^−1^, NH_3_/NH${}_{4}^{+}$ <0.1 mg L^−1^. Plastic mesh was used as artificial substrate (Baeza-Rojano et al., 2013a). Amphipods were fed daily with a commercial feed (Wardley® Tropical Fish Flake Food, USA). Amphipod species were identified using taxonomic keys (Shoemaker, 1956; Vader & Krapp-Schickel, 2012) and validated by Dr. Carlos E. Paz-Ríos, a local peracarid expert.

### Biometry

From the initial, pooled, collection of amphipods, a sample of 100 individuals of each species were randomly collected using an aquarium net, and the total length, wet weight, dry weight and water content were determined for each individual. The total length was defined as the length along the dorsal edge, from the tip of the rostrum to the telson tip. Measurements were made using photographs taken with a digital camera (Summit SK2-5.2X, OptixCam, VA, USA) coupled with a stereo microscope (Nikon SMZ800N, Nikon, Tokyo, Japan). For weight measurements, amphipods were blotted briefly on filter paper and then weighed with an OHAUS Explorer analytical balance (NJ, USA; precision 0.1 mg). For the dry weight measurement, amphipods were placed in preweighed capsules and dried at 60 °C for at least 24 h until the weight was constant. Sex was determined in adult specimens which, unlike juveniles, show sexual dimorphism, in agreement with that reported by Johnson, Stevens & Watling (2001) for other amphipod species. Females were identified based on the presence of brood pouch and males based on the body size and the size of the second gnathopod which is enlarged in males (Johnson, Stevens & Watling, 2001).

### Gross energy content

To obtain the gross energy content, six replicate samples (10 g wet weight per sample) of each amphipod species were randomly collected from the initial collection of amphipods. Samples were dried (24 h at 65 °C), homogenized and then analyzed using an Isoperibol Calorimeter (Parr Inst. Co., Moline, IL) according to the Association of Official Analytical Chemists (AOAC International, 1998). For comparative purposes, six samples of *Artemia* adult (INVE, Belgium) enriched with krill oil (Aceite de Krill Biogrow®, PROAQUA, SIN, MX) were also included in the analysis.

### Generation time

To address generation time, gravid females of each species were collected from the stocking culture tanks, and newly released juveniles from brood pouch were cultured until they first entered precopulatory pairing (precopula). This parameter is commonly used, and a reliable indicator of sexual maturity in species that display this behavior (Galipaud et al., 2015; Tsoi & Chu, 2005). Particularly, juveniles were placed in 15 experimental tanks (1 L) in groups of approximately 16 individuals, depending on availability and batch size (individuals within the same tank of the same age). They were fed a shrimp diet (Api Camarón® Intensivo, MaltaCleyton, YU, MEX) once a day to apparent satiation. Complete water replacements were performed daily and water in the tanks was maintained at 24.0 ± 0.5 °C, salinity 37  ± 1 ppt, pH 8.1–8.3, NO${}_{2}^{-}$ <0.3 mg L^−1^, NO${}_{3}^{-}$ <5 mg L^−1^, NH_3_/NH${}_{4}^{+}$ <0.1 mg L^−1^, without aeration. When a pair entered precopula, the age (days of culture) and the size of each individual in the pair were recorded and then the animals removed from the experimental tank, to avoid quantifying the same specimens twice. One hundred and five pairs entered precopula in the case of *P. hawaiensis*, whereas none in the case of *E. pectenicrus*. As the latter did not show precopula, it was excluded from further experiments that involved reproduction (e.i. generation time, cannibalism, effect of diet on fecundity and juvenile growth), except for the experiment on the effect of sex ratio on fecundity (‘Effect of sex ratio on fecundity’).

### Sex ratio

In order to know the sex ratio of amphipod populations occurring in the aquaculture systems, a sample of 3,033 adults of *P. hawaiensis* and another of 1,168 adults of *E. pectenicrus* were randomly taken from the initial collection of amphipods and sex was determined as described in ‘Biometry’. Sample size differed between species due to the lower availability of collected *E. pectenicrus* specimens, as noted in ‘Amphipod collection and maintenance’.

### Fecundity

From the initial collection of amphipods, females were isolated (*P. hawaiensis n* = 93 and *E. pectenicrus n* = 65) and the total length, as well as the number of embryos in each female’s brood pouch, was recorded. As *E. pectenicrus* showed parental care (hatched individuals were observed in brood pouch) (preliminary observations), females showing this behavior were not used for fecundity analysis, to make reliable comparisons. Total length was measured as described in ‘Biometry’ and the embryos were counted using a stereo microscope (Nikon SMZ800, Nikon, Tokyo, Japan).

### Pilot culture

A pilot culture of *P. hawaiensis* and another of *E. pectenicrus* were performed to estimate the net production of juveniles by a given starting population of adults. For each experiment, 225 precopula pairs were used to ensure their readiness to copulate and reproductive competence. They were collected from the initial stock and then randomly assigned to 15 tanks of 9.4 L (15 couples per tank) connected to a closed recirculation system as in Calado et al. (2008). In order to reduce cannibalism in the tanks, 4 cement hand-made rocks of approximately 100 mm diameter and 5 mm thick wrapped with plastic mesh (Baeza-Rojano et al., 2013a) were introduced in the tanks. Animals were fed daily to apparent satiation with a commercial feed designed for omnivorous marine fish (New Life Spectrum® Marine Fish Formula, New Life International, FL, USA) for 30 days. Before each feeding, feces and uneaten food were siphoned out daily and water chemical-physical parameters were kept as in ‘Amphipod collection and maintenance’. The number of juveniles produced after 15 and 30 days was determined.

### Effect of sex ratio on fecundity

To determine whether manipulating sex ratio could be a useful tool to increase the production of both amphipod species, the effect of a female- and male-biased sex ratio, and a control group (8m:2f, 2m:8f, 5m:5f, respectively), on the fecundity was assessed. As in ‘Pilot culture’, precopula pairs collected from the initial stock were used to ensure their readiness to copulate and reproductive competence. In these experiments, 10 adults (either 8m:2f, 5m:5f or 2m:8f) in each amphipod species were randomly placed in small tanks (0.22 L) (4 replicate tanks per experimental group) provided with plastic mesh as substratum and fed a commercial fish feed (Wardley® Tropical Fish Flake Food, USA) daily to apparent satiation. Complete water replacements were performed daily and chemical-physical parameters were kept as in ‘Generation time’, except that a slightly higher variation in water temperature was recorded (24 ± 2 °C). During water replacement (daily), the number of juveniles released per tank was registered and if any adult died, it was immediately replaced with a brooding adult to keep the sex ratio constant.

### Cannibalism

For this experiment, both juveniles and adults of *P. hawaiensis* were obtained from animals harvested from the pilot culture (‘Pilot culture’). Ten juveniles (15 days post-release) were placed together with two adults (either male or female) in small tanks (120 mL) (10 tanks in total) without artificial substratum (5 replicate tanks of males and 5 of females). They were fed (Wardley® Tropical Fish Flake Food, USA) ad libitum twice a day for 10 days. Good water quality (same chemical-physical parameters as in ‘Effect of sex ratio on fecundity’) was achieved by complete water replacements performed daily. The number of remaining juveniles was recorded at the end of the experiment.

### Effect of diet on the fecundity of *P. hawaiensis*

To evaluate the effect of diet on the fecundity of *P. hawaiensis*, three different kinds of diets were used. These were a typical commercial shrimp diet (Diet 1) (Api Camarón® Intensivo, MaltaCleyton, YU, MEX), a plant-based diet (40% protein) containing phytase and designed for shrimp nutrition at the UMDI-Sisal, UNAM (Diet 2), and a commercial tropical fish diet (Diet 3) (Wardley® Tropical Fish Flake Food, USA). For this experiment, precopula pairs were randomly collected from the stocking culture tanks, using an aquarium net, and then 2 males and 8 non-ovigerous females were randomly selected and placed in 1 L tanks provided with plastic mesh. Non-ovigerous females were used to control for stage of development. Animals were fed to apparent satiation for 29 days (5 replicates per dietary treatment). Complete water replacements were performed daily, and water quality parameters fluctuated as in ‘Cannibalism’. The number of juveniles released per tank was recorded every other day.

### Effect of diet on the growth of *P. hawaiensis*

The same three diets from the previous experiment (‘Effect of diet on the fecundity of *P. hawaiensis’*) and the same rearing conditions, except that without the adults and using smaller tanks (0.22 L), were applied to assess the effect of diet on the juvenile growth. Particularly, 20 newly released juveniles per dietary treatment were individually placed in small tanks (0.22 L) and fed to apparent satiation every other day for 30 days. A negative control group (without feeding) was included in the analysis. The total length was measured at days 0, 10, 20 and 30 using photographs as described in ‘Biometry’.

### Statistical analysis

Total length data were examined by frequency distribution plots. Differences between groups (total length, wet weight, dry weight, fecundity, size of first precopula and cannibalism) were tested by t Student test (one-tail) (Table 1). Differences among groups (gross energy content, effect of sex ratio on fecundity, and effect of diet on fecundity and on growth at the end of the experiment) were tested by means of one-way ANOVA, followed by Tukey’s tests (Table 1). The relationship between wet weight and total length and between dry weight and wet weight, as well as between fecundity and total length, were analyzed using linear regressions (Table 1). Data were previously transformed (natural logarithm) to better meet normality and homoscedasticity. Normality and homoscedasticity for all tests were verified through residual plots according to Zuur, Leno & Smith (2007). The statistical software package Prism5 (GraphPad Software) was used; significance was set at *p* < 0.05. All results are presented as mean ± SD.

**Table 1:** Statistical analyses performed and summary results.

Analysis	Comparison	Statistic	Probability	Degrees of freedom	Transform
One-tailed t test	Total length by species	*t* = 7.9	<0.0001	195	–
	Total length by sex (*P. hawaiensis*)	*t* = 3.6	=0.0002	98	–
	Total length by sex (*E. pectenicrus*)	*t* = 3.6	=0.0003	95	–
	Wet weight by species	*t* = 7.9	<0.0001	194	–
	Dry weight by species	*t* = 8.7	<0.0001	194	–
	Wet weight by sex (*P. hawaiensis*)	*t* = 5.0	<0.0001	98	–
	Wet weight by sex (*E. pectenicrus*)	*t* = 5.2	<0.0001	95	–
	Dry weight by sex (*P. hawaiensis*)	*t* = 5.3	<0.0001	98	
	Dry weight by sex (*E. pectenicrus*)	*t* = 5.1	<0.0001	95	
	Size of first precopula by sex (*P. hawaiensis*)	*t* = 11.28	<0.0001	208	–
	Fecundity by species	*t* = 7.1	<0.0001	143	–
	Cannibalism by sex (*P. hawaiensis*)	*t* = 2.2	=0.0298	8	–
One-way ANOVA	Gross energy content	*F* = 85.68	<0.0001	2, 12	–
	Effect of sex ratio on fecundity (*P. hawaiensis*)	*F* = 14.4	=0.0016	2, 9	Y = Ln(Y)
	Effect of sex ratio on fecundity (*E. pectenicrus*)	*F* = 2.6	=0.1286	2, 9	–
	Effect of diet on fecundity (*P. hawaiensis*)	*F* = 0.6	=0.5572	2, 12	–
	Effect of diet on total length at day 30 (*P. hawaiensis*)	*F* = 3.3	=0.0461	2, 51	–
Regression analysis	ln Wet weight vs ln Total length (*P. hawaiensis*)	*F* = 656.8	<0.0001	1, 97	X = Ln(X); Y = Ln(Y)
	ln Wet weight vs ln Total length (*E. pectenicrus*)	*F* = 552.7	<0.0001	1, 95	X = Ln(X); Y = Ln(Y)
	Wet weight vs dry weight (*P. hawaiensis*)	*F* = 1988	<0.0001	1, 97	
	Wet weight vs dry weight (*E. pectenicrus*)	*F* = 3795	<0.0001	1,95	
	Fecundity vs total length (*P. hawaiensis*)	*F* = 40.3	<0.0001	1, 91	
	Fecundity vs total length (*E. pectenicrus*)	*F* = 3.7	=0.0611	1, 50	

## Results

### Biometry

**Figure 1:**
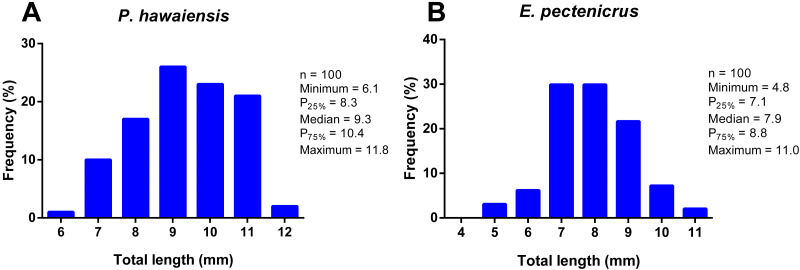
Total length frequency distribution of *P. hawaiensis* (A; *n* = 100) and *E. pectenicrus* (B; *n* = 97).

**Figure 2:**
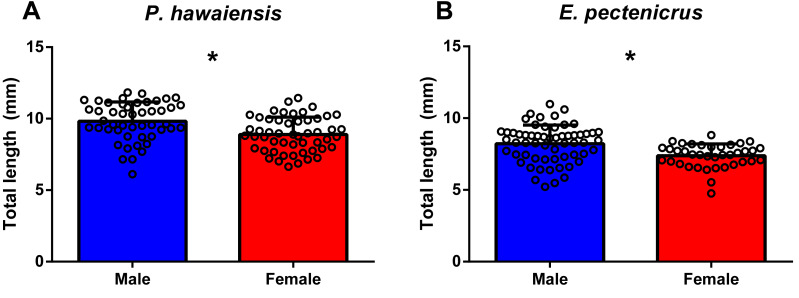
Total length of males and females of *P. hawaiensis* (A; *n* = 100) and *E. pectenicrus* (B; *n* = 97). Asterisk indicate statistically significant differences between groups (*p* < 0.05).

The total length frequency distribution of *P. hawaiensis* and *E. pectenicrus* are given in Fig. 1. *P. hawaiensis* showed a significantly higher (*p* < 10^−4^) total length (9.3 ± 1.3 mm) with respect to *E. pectenicrus* (7.9 ± 1.2 mm). Similarly, the maximum (11.83 mm) and minimum (6.12 mm) total length of *P. hawaiensis* reached higher values with respect to *E. pectenicrus* (10.98 mm and 4.75 mm, respectively). Sorted by sex, males of both *P. hawaiensis* (9.8 ± 1.3 mm) and *E. pectenicrus* (8.2 ± 1.3 mm) showed a significantly higher (*p* = 20^−4^, *p* = 30^−4^, respectively) total length with respect to females (8.9 ± and 7.4 ± 0.8, respectively) (Fig. 2).

### Wet and dry weight

**Figure 3:**
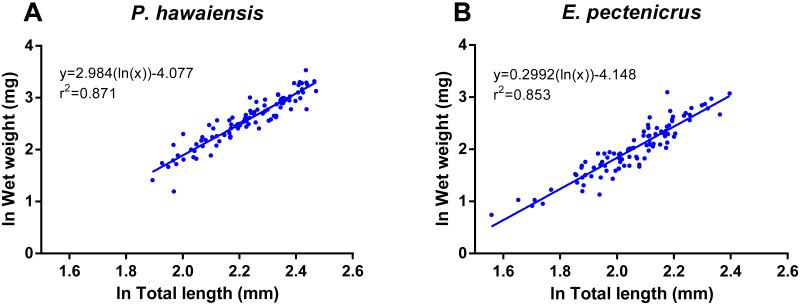
Relationship between ln wet weight and ln total length of *P. hawaiensis* (A; *n* = 99) and *E. pectenicrus* (B; *n* = 97).

**Figure 4:**
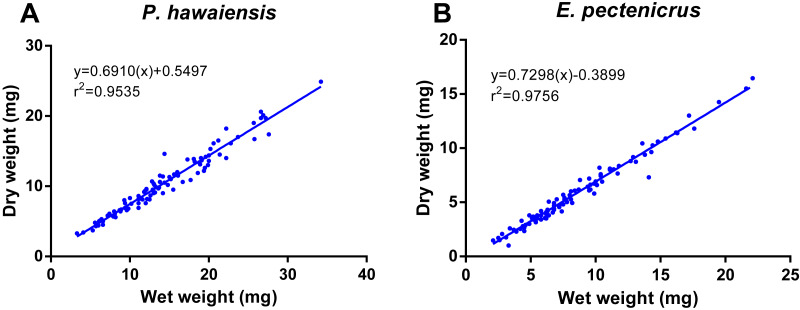
Relationship between dry weight and wet weight of *P. hawaiensis* (A; *n* = 97) and *E. pectenicrus* (B; *n* = 97).

A significant relationship (*p* < 10^−4^) was observed between ln wet weight and ln total length for both *P. hawaiensis* (*r*^2^ = 0.871) and *E. pectenicrus* (*r*^2^ = 0.853) (Fig. 3). *P. hawaiensis* showed a significantly higher (*p* < 10^−4^) wet weight (14.4 ± 6.2 mg, 73.9 ± 7.1% water content) and dry weight (10.5 ± 4.4 mg) with respect to *E. pectenicrus* (wet weight 8.4 ± 4.3 mg, 66.7 ± 13.5% water content; dry weight 5.7 ± 3.2 mg). Sorted by sex, males of both *P. hawaiensis* and *E. pectenicrus* showed a significantly higher (*p* < 10^−4^) wet weight (17.6 ± 6.5 mg and 10.1 ± 4.8 mg, respectively) and dry weight (12.6 ± 5.0 mg and 6.9 ± 3.4 mg) with respect to females (wet weight 11.5 ± 4.1 mg and 5.8 ± 1.8 mg, respectively; dry weight 8.4 ± 2.9 mg and 3.9 ± 1.5 mg, respectively). In regard to the relationship between wet weight and dry weight (Fig. 4), a significant (*p* < 10^−4^) relationship was observed for both *P. hawaiensis* (*r*^2^ = 0.9535) and *E. pectenicrus* (*r*^2^ = 0.9756).

### Gross energy content

Regarding gross energy content (dry matter basis), significant differences (*p* < 10^−4^) were observed among *P. hawaiensis*, *E. pectenicrus* and enriched *Artemia*. In particular, *P. hawaiensis* (16.71 ± 0.67 kJ g^−1^) showed a significantly higher (*p* < 0.05) gross energy content with respect to *E. pectenicrus* (12.86 ± 0.82 kJ g^−1^), while enriched *Artemia* (19.98 ± 1.12 kJ g^−1^) showed a significantly higher (*p* < 0.05) energy content with respect to both amphipod species.

### Generation time

The time of first precopula, as an indicator of sexual maturity and thus generation time, occurred 50.9 ± 5.8 days after hatching in *P. hawaiensis*, with significant differences (*p* < 10^−4^) in size between sexes. In particular, due to a higher growth rate observed in males, males showed (7.5 ± 0.5 mm) a significantly higher (*p* < 10^−4^) total length with respect to females (6.7 ± 0.5 mm) at first precopula. Regarding *E. pectenicrus*, the animals did not form pairs and thus the time required to reach sexual maturity could not be determined.

### Sex ratio

*P. hawaiensis* showed a sex ratio (females/males) of 2.24, whereas *E. pectenicrus* displayed a ratio of 1.34.

### Fecundity

**Figure 5:**
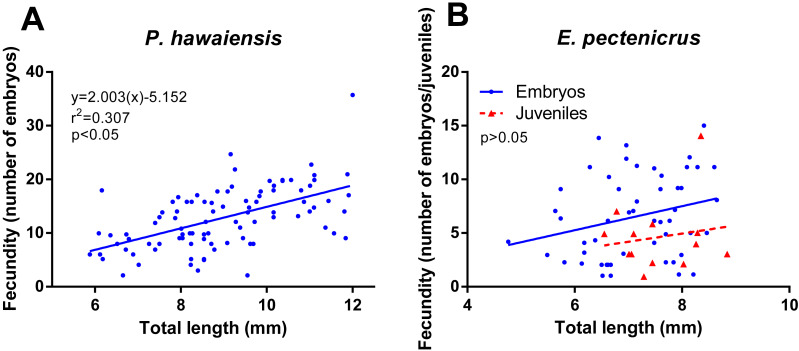
Relationship between fecundity and total length of *P. hawaiensis* (A; *n* = 93) and *E. pectenicrus* (B; *n* = 52). Hatched Juveniles (*n* = 13) were found in *E. pectenicrus* brood pouch as indicated by the legend.

Concerning fecundity, a high variation for both amphipods species was observed but with a significant relationship (*p* < 10^−4^) between fecundity and the female’s size in *P. hawaiensis* (*r*^2^ = 0.307) (Fig. 5A). A similar relationship was observed in *E. pectenicrus* (Fig. 5B), but was no statistically significant (*p* = 0.061). *P. hawaiensis* (12.8 ± 5.7 embryos per female) showed a significantly higher (*p* < 10^−4^) fecundity with respect to *E. pectenicrus* (6.5 ± 3.9 embryos per female). Interestingly, hatched individuals were found in the brood pouch of *E. pectenicrus* (4.6 ± 3.3 individuals) (Fig. 5B), but not in that of *P. hawaiensis*.

### Pilot culture

After 15 days of culture, tanks containing 15 amphipod couples produced 73 ± 16 juveniles per tank for *P. hawaiensis*. After the next 15 days of culture, the same tanks produced 89 ± 20 juveniles per tank. Considering all 30 experimental days, this corresponded to an average of 0.36 ± 0.08 juveniles per couple per day. Regarding *E. pectenicrus,* no juveniles were produced by the couples during the whole experimental period, except for one tank that produced 5 juveniles per couple.

### Effect of sex ratio on fecundity

**Figure 6:**
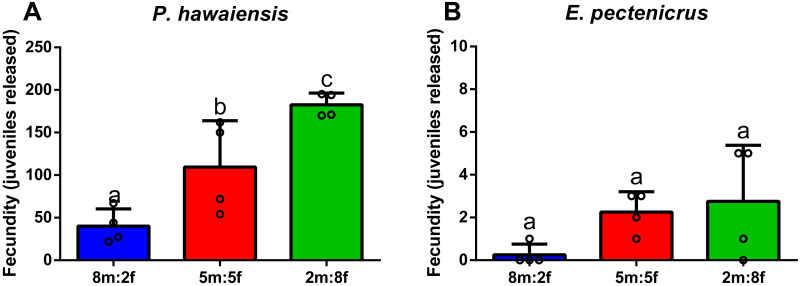
Effect of sex ratio (males [m]: females [f]) on the fecundity of *P. hawaiensis* (A; *n* = 4) and *E. pectenicrus* (B; *n* = 4). Different letters indicate statistically significant differences among the experimental groups (*p* < 0.05).

Regarding the effect of sex ratio on the fecundity of *P. hawaiensis* (Fig. 6A), significant differences (*p* = 0.002) were observed among groups, with fecundity (number of juveniles released per group) significantly increasing with the increase of females in relation to males. In particular, 8m:2f ratio (40 ± 20.3 juveniles) produced a significantly lower (*p* < 0.05) fecundity with respect to both 5m:5f (109.5 ± 54.4 juveniles) and 2m:8f (182.5 ± 13.9 juveniles) ratios. In addition, personal observations (H. I. López-Vázquez, 2015) indicated a high competition between males at 8m:2f ratio and this resulted in higher mortalities for males (not recorded). Regarding *E. pectenicrus,* an extremely low fecundity was detected (8m:2f ratio 0.3 ± 0.5, 5m:5f 2.3 ± 1 and 2m:8f 2.8 ± 2.6), without significant differences (*p* = 0.129) among groups (Fig. 6B).

### Cannibalism in P. hawaiensis

Regarding cannibalism in *P. hawaiensis*, both males and females showed a high cannibalistic behavior, with males (8.4 ± 0.9 juveniles eaten out of 10 per male) showing a significantly higher (*p* = 0.029) cannibalistic behavior with respect to females (6.6 ± 1.6 juveniles eaten out of 10 per female).

### Effect of diet on the fecundity of P. hawaiensis

**Figure 7:**
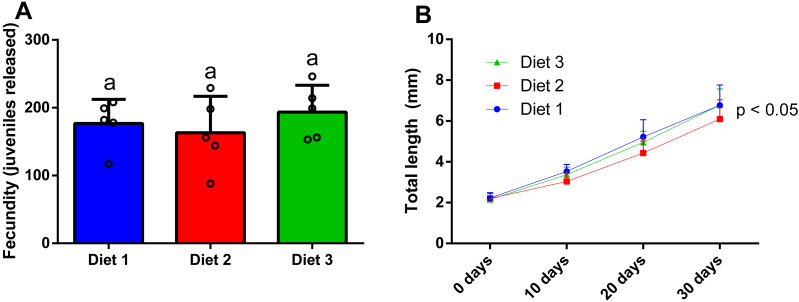
Effect of diet on the fecundity (A) (two males and eight females per replicate) and the juvenile growth (*n* = 20) of *P. hawaiensis* (B). Diet 1: shrimp diet; Diet 2: plant-based diet; Diet 3: commercial tropical fish diet. No significant differences (*p* > 0.05) were observed among the experimental groups in A; *p* < 0.05 in B indicate that significant differences were observed at 30 days.

After 28 days of dietary treatment, at sex ratio of 2m:8f, no significant differences (*p* = 0.557) were observed among dietary treatments (Fig. 7A). In particular, amphipods fed on a shrimp diet (Diet 1) produced 176.8 ± 35.6 juveniles, while amphipods fed on a plant-based diet (Diet 2) produced 163 ± 53.9 juveniles and amphipods fed on a commercial tropical fish diet (Diet 3) produced 193.6 ± 39.5 juveniles.

### Effect of diet on the growth of P. hawaiensis

After 30 days of dietary treatment, all juveniles showed a roughly three-fold increase in total length, with slight but significant differences (*p* = 0.046) among groups (Fig. 7B). In particular, at the end of the experiment, Diet 2 (3.51 ± 1.8 mm) showed a lower total length with respect to Diet 1 and 2 (4.5 ± 2.13 and 4.23 ± 1.8 mm, respectively).

## Discussion

### General results

Marine amphipods have been recently recognized as natural food organisms alternative to traditional live food such as *Artemia* (Woods, 2009; Jiménez-Prada et al., 2018). These are a diverse group of benthonic crustaceans commonly found forming dense colonies in natural or artificial aquatic habitats (Lourido, Moreira & Troncoso, 2008; Vázquez-Luis, Sanchez-Jerez & Bayle-Sempere, 2013). They are rich in essential fatty acids such as DHA and EPA, can feed on a variety of food including detritus, and can tolerate wide fluctuations of chemical-physical parameters, thus interesting candidates for aquaculture (Guerra-García, Martínez-Pita & Pita, 2004; Fernandez-Gonzalez et al., 2018; Vargas-Abúndez, Simões & Mascaró, 2018). The present study explored the aquaculture potential of *P. hawaiensis* and *E. pectenicrus*, two cosmopolitan marine species that naturally proliferate in research facilities, displaying qualities desirable for aquaculture propagation.

Results on the population and reproductive biology, gross energy content, generation time, amphipod productivity and dietary effects on fecundity and juvenile growth indicated that *P. hawaiensis,* unlike *E. pectenicrus,* could be a suitable candidate for aquaculture, despite quite long generation times and relatively low fecundity levels. *P. hawaiensis* was easily kept in laboratory conditions, fed on different diets without compromising fecundity and reproduced continuously at an estimated rate of 0.36 ± 0.08 juveniles per couple per day. Compared to one of the most productive amphipod species known to date, *C. scaura,* that reproduced in large tanks (100 L) at a calculated rate of 1.09 juveniles per couple per day (Baeza-Rojano et al., 2013a), productivity values for *P. hawaiensis* were lower, one third approximately. Although more studies are needed to optimize the *P. hawaiensis* culture and to explore medium- and large-scale production, production numbers obtained in the present study may be sufficient to sustain the aquaculture of species that require low food quantities such as seahorses and other highly-priced marine ornamental organisms, where food quality is more important than food quantity at the lowest cost possible.

### Biometry/Wet weight and dry weight

Size of the most commonly used live preys in larviculture, including *Artemia*, rotifers and copepods, range approximately from 60 to 500 µm, depending on the species and developmental stage (Van Stappen, 1996; Støttrup & McEvoy, 2003). Amphipods, with their larger size, far from being too big, may represent a new live prey alternative to adult *Artemia*. They could be used to feed a wide spectrum of species, including early juveniles of large species, such as cuttlefish (*Sepia officinalis)* and Yucatan octopus (*Octopus maya)*, whose early rearing relies mainly on live prey (Baeza-Rojano et al., 2010; Baeza-Rojano et al., 2013b; Wang & Jeffs, 2014; Planas et al., 2017), or matured juveniles and adults of small species, such as seahorses and other ornamental fish. Accordingly, average-sized amphipods, ranging 6.1–8.7 mm, 2–6 mm and 7–8 mm have been successfully used to feed juvenile, subadult and adult seahorses (Murugan et al., 2009; Vargas-Abúndez, Simões & Mascaró, 2018), octopus hatchlings (Baeza-Rojano et al., 2013b) and cuttlefish hatchlings (Baeza-Rojano et al., 2010), respectively. For potential use in other species, it will be important to consider the appropriate ratio of prey size to mouth gape size (usually <0.5) in fish that swallow the entire prey (Shirota, 1970; Cunha & Planas, 1999). Of less importance, it will be in larvae/juveniles with developed teeth or mechanical structures that aid grasping, biting or scratching feed particles such as parrotfish and octopus (Bone & Moore, 2008; Baeza-Rojano et al., 2013b), or if amphipods are incorporated as amphipod meal in compound feeds (Harlıoğlu & Farhadi, 2018). For this latter possible application, it is especially important to address the nutritional value of *P. hawaiensis*.

An interesting characteristic observed in both amphipod species studied, with potential applications for their aquaculture management, was the evident sexual dimorphism (Figs. 2A and 2B). Males from both amphipod species showed a significantly larger size (length and weight) with respect to females and sex was clearly distinguished based on both the body size and the size of the second gnathopod, which were enlarged in males, as expected. This sexual dimorphism is critical for reproductive success and is common among amphipods and isopods (Johnson, Stevens & Watling, 2001). Since females are receptive to fertilization shortly after molting and only for a short time period, males grab and hold females for a few hours up to several days to ensure copulation over competitors (Galipaud et al., 2015). As a result, reproductive individuals can be seen paired (precopula) over the culture tank. Thus, amphipods of the largest sizes can be sorted by sex, for example, using nets of appropriate size or simply manually (as sex of adult individuals can be distinguished by the naked eye). This can facilitate the manipulation of sex ratio for culture management.

### Gross energy content

Providing adequate energy levels through the diet is essential for proper animal development and growth (Izquierdo, 1996). Both amphipod species showed valuable levels of gross energy, that were comparable to enriched *Artemia*. Lipids are the macronutrient with the highest energy content and thus the high energy levels found in the enriched *Artemia* could be associated with the high-energy content of the krill oil provided as enrichment. Amphipods naturally have a high lipid content, particularly of EPA and DHA (Guerra-García, Martínez-Pita & Pita, 2004; Guerra-García et al., 2016; Cook et al., 2010), and these levels could be comparable to those found in Antarctic krill (Kolanowski, Stolyhwo & Grabowski, 2007). Again, it remains to be assessed the biochemical composition of the amphipod species studied to confirm their nutritional value.

### Sex ratio

It is known that sex ratio of amphipod populations may vary according to a number of factors, including food availability, predation, season, difference in longevity between sexes and period of time females are receptive to fertilization (Sheader, 1983). Among these, it is plausible that the higher proportion of females observed in the aquaculture systems were due to the competition between males. In most gammarids, the low availability of females, due to the low time they are receptive to fertilization, induce an intense competition between males that often leads to death (Johnson, Stevens & Watling, 2001). More information is needed to better understand how sex ratio may fluctuate throughout the year and apply this knowledge to maximize amphipod production.

### Fecundity and generation time

Marsupial volume is usually correlated to female size and thus tend to have larger broods (Johnson, Stevens & Watling, 2001). This may explain, at least partially, the higher fecundity in *P. hawaiensis* with respect to *E. pectenicrus*, as *P. hawaiensis* are significantly larger than *E. pectenicrus*. Similar results have been reported for *P. hawaiensis*, with fecundity ranging from 4 to 21 embryos per female (Lee & Nicol, 1980). For *E. pectenicrus*, to the best of our knowledge, no previous information is available, but for a closely related species, *Elasmopus levis*, 22.3 ± 2.1 (standard error) embryos per female were reported, which is over a three-fold difference (Borowsky, 1986). The fecundity values obtained are lower than those reported for cultured (36.3 eggs/embryos per female in *C. scaura* in average) (Baeza-Rojano et al., 2013a) and wild caprellids (from 2 to 55 in *Pseudaeginella montoucheti* and from 2 to 13 eggs in *P. tenuis*) (Garcia, Cunha & Jacobucci, 2019). Caprellids, unlike gammarids, show a relatively sedentary behavior (low energy expenditure) that could explain their higher fecundity; however, the active behavior of gammarids could be advantageous stimulating feeding responses, as previously observed when feeding *S. officinalis* hatchlings (Baeza-Rojano et al., 2010).

In assessing reproductive potential and planning culture strategies, another important aspect to consider is generation time, or in other words, the time needed for a hatchling to become sexually mature and thus to close its life cycle. Generally, the faster, the better. *P. hawaiensis* reached sexual maturity after 50.9 ± 5.8 days of culture. Due to a higher growth rate, males showed a higher length and weight at sexual maturity than females, as commonly observed in other mate guarding amphipods (Othman & Pascoe, 2001). Accordingly, the review by Sun & Patel (2019) mentioned that *P. hawaiensis* become sexually mature after two months, although Artal et al. (2018) reported three months for this species to reach sexual maturity. Generally, growth rate and development are positively correlated with water temperature (Tsoi, Chiu & Chu, 2005). The study by Artal et al. (2018) used the same water temperature as the present one but with a higher variation (± 2 °C vs in contrast to 0.5 °C). Thus, this higher variation may explain the differences observed. The present information is useful to plan aspects of amphipod culture, such as harvesting strategy. For example, given the almost two-month period needed for *P. hawaiensis* to mature, culture operations should last at least two months after “seeding” to allow hatchlings to mature and have the highest biomass. In agreement with the present recommendation, the mass culture of *C. scaura* lasted three months and showed a 50 fold increase of initial population seeding (Baeza-Rojano et al., 2013a). In alternative, a culture stock from which regular harvesting, weekly for example, can be performed, as females can produce eggs every two weeks (Sun & Patel, 2019). For a specific and maximum harvesting, further culture experiments need to be performed.

Regarding generation time in *E. pectenicrus*, animals did not form precopula pairs and thus the time required to reach sexual maturity could not be determined. Some amphipod species such as *E. levis* do not exhibit precopulatory activity (Borowsky, 1986) and this could be the case for this species. Alternatively, it is also possible that precopula in *E. pectenicrus* is very brief and passed unnoticed despite careful observation, or simply that laboratory conditions were not adequate for the propagation of this species, since animals showed a good growth rate (not recorded) but did not form pairs. The feeding habits of *E. pectenicrus* are unknown, but this species was collected from filamentous algae mats, which could suggest that it is strictly herbivorous. Thus, the different diets used in the present study might not be completely adequate for this species. In addition, it is possible that laboratory conditions inhibited reproduction via lack or presence of environmental clues such as photoperiod or salinity that trigger or inhibit reproduction (Borowsky, 1986). A possible inhibiting factor could have been the absence of sediment in the experimental containers, as individuals from the genus *Elasmopus* usually build nests where mating takes place (I. Winfield, 2015, pers. com.). In the experimental tanks of the present study artificial substrate was introduced but this could have been inappropriate for this species. More information on the feeding habits and the reproductive biology of *E. pectenicrus* are needed to better assess the potential of this species for aquaculture. For this reason, *E. pectenicrus* was excluded from experiments aimed at optimizing the culture.

In spite of the relatively low fecundity and quite long generation times, surprisingly, amphipods are often found in high numbers in their natural habitats (>100,000 individuals m^2^) (Oliva-Rivera, 2003; Lourido, Moreira & Troncoso, 2008; Vázquez-Luis, Sanchez-Jerez & Bayle-Sempere, 2013), where they can be one of the major components of the benthos, as it is the case for the two species analyzed (>7,000 individuals m^2^) (Zakhama-Sraieb & Charfi-Cheikhrouha, 2010; Paz-Ríos, Simões & Ardisson, 2013). In addition, a growing body of evidence indicates that amphipods can proliferate in artificial habitats such as in biofouling formations (Fernández-González, 2017), offshore (Fernandez-Gonzalez et al., 2018) and inland aquaculture facilities (González, Pérez-Schultheiss & López, 2011). Thus, it appears that such low reproduction rates are effectively compensated by other adaptations that facilitate their proliferation. One of these is related to the peculiar reproductive biology of amphipods; peracarids, including *P. hawaiensis* and *E. pectenicrus*, do not have any (true) larval stage (direct larval development) and the embryos are rather carried in the female’s brood pouch where they hatch and emerge as miniature versions of the adult (Browne et al., 2005). As a result, offsprings are less vulnerable to predation and their survival rates are dramatically increased in comparison with species displaying larval development. In addition, some species exhibit various degrees of parental care that can be further advantageous for their survival and access to food (Moore, 1981). In both studied species, developing embryos were found in the female’s brood pouch and, in addition, *E. pectenicrus* showed a possible parental care behavior, since completely developed juveniles were observed being carried by females within the marsupium (Fig. 5B). This reproductive strategy not only confers advantages for survival in the wild and potentially in aquaculture facilities, but it also means that no special feeding requirements are needed to raise the juveniles nor any particular holding system, as they can be reared in the same holding system as the adults without compromising growth and survival.

### Pilot culture

The pilot culture of *P. hawaiensis* revealed a productivity rate of 0.36 ± 0.08 juveniles per couple per day. If such productivity is multiplied by the average amphipod wet weight (14.4 ± 6.2 mg), this would eventually (in around two months) give a yield of 2.33 g of amphipod per tank (15 amphipod couples). In comparison with one of the most promising amphipods with potential in aquaculture known to date, *C. scaura* (Baeza-Rojano et al., 2013a)*,* this productivity level was lower, one third approximately of the *C. scaura* production. In the preliminary culture of *C. scaura* in big outdoor tanks (100 L) by Baeza-Rojano et al. (2013a), starting from 125 females and 125 males per tank, after 3 months, a final mean population size of 12,510 ± 2,200 individuals/tank was obtained, a 50-fold increase of the initial population. It corresponds to 1.09 juveniles per couple per day. In another study, where the use of collector devices (5 L) that were introduced into floating cages for European sea bass (*Dicentrarchus labrax*) and gilthead sea bream (*Sparus aurata*) of an off-shore farm, monthly production was estimated in 10 g wet-weight per collector. Therefore, the productivity of *P. hawaiensis* is lower to that reported previously for other species, yet comparable. Further studies are needed to evaluate whether an optimized mass culture can give similar or even improved yields.

### Effect of sex ratio on fecundity

Results on the in situ fecundity and the effect of sex ratio on fecundity suggested, for the first time, that this is a key factor for culture optimization. Considering that fecundity of *P. hawaiensis* was 12 ± 7.8 embryos per female and that they may reproduce almost continuously, every 15 days (Sun & Patel, 2019), yields for this species could reach up to 0.8 juveniles per couple per day, almost comparable to caprellid yields (Woods, 2009; Guerra-García et al., 2016). In addition, fecundity per tank (0.22 L) significatively increased from 40 ± 20.3 juveniles to 182.5 ± 13.9 by increasing sex ratio towards females, from 8m:2f to 2m:8f, pointing out the importance of manipulating sex ratio for production optimization. In the tanks with a high proportion of males (8m:2f), not only fecundity decreased significatively, but a high competition among males was observed (H. I. López-Vázquez, 2015, personal observations) and this resulted in a higher mortality rate (not quantified). Taking advantage of the larger size of the males of this species, a practical way to sort the amphipods by sex for culture seeding could be the use of nets of appropriate size, as discussed above.

### Effect of diet on fecundity and juvenile growth of *P. hawaiensis*

*P. hawaiensis* are detritivores, thus may potentially feed on a wide variety of foods, including decaying organic material and diets composed of inexpensive ingredients (Shoemaker, 1956; Poovachiranon, Boto & Duke, 1986). In the present study, three different kinds of diets were evaluated and highlighted the potential of *P. hawaiensis* to successfully grow using a variety of feeds without negative impacts on fecundity. The present results are promising and open the possibility to use a wide range of protein sources, including cheap proteins, and avoiding expensive ingredients such as fish meal, with a great potential on reducing production costs. However, the biochemical composition of *P. hawaiensis* was not addressed in the present study, except for gross energy content. Marine, wild amphipods generally present biochemical profiles that match nutritional requirements of marine fish, namely in terms of essential fatty acids, such as EPA and DHA (Woods, 2009; Baeza-Rojano, Hachero-Cruzado & Guerra-García, 2014), but shifting their natural diet using cheap, non-marine ingredients could alter their nutritional value (Cook et al., 2010). There is presently limited information to fully understand how this shifting could affect nutritional values. Particularly, it has been suggested that two gammarid species may have the capacity to synthesize substantial amounts of long-chain PUFAs (LC-PUFAs) starting from shorter precursors (C18). Taking advantage of this, it was suggested that diets of terrestrial origin (lupin meal and carrot leaves) could be used as amphipod feed without negatively impacting the fatty acid composition in comparison with two marine diets *(sea lettuces Ulva* spp. and brown algae *Fucus* spp.) (Alberts-Hubatsch, Slater & Beermann, 2019). In another study, caprellids were fed on a diet of detritus from an aquaculture farm, and this resulted in excellent fatty acid profiles, comparable to those obtained when fed on phytoplankton (Guerra-García et al., 2016). Whether cheap feed materials, other than aquaculture feed residues, can be used as a viable food source for amphipod aquaculture requires further research. Nevertheless, with their great dietary plasticity and environmental tolerance, *P. hawaiensis* could become a valid candidate for massive production in self-producing biofloc systems.

### Cannibalism

One point of caution in the amphipod aquaculture was highlighted by a further experiment that revealed a high cannibalistic behavior upon the juveniles by both males and females of *P. hawaiensis* even in the presence of plenty of food. In the test for cannibalism, however, no artificial substrate was introduced in the experimental tanks (details in ‘Cannibalism’), and this was probably inadequate for providing hiding for the juveniles. Therefore, studies are needed to address the possible negative impact of cannibalism on the commercial scale for amphipod production as well as ways to reduce it. A key factor to reduce it will probably be the use of natural or artificial substrates that maximize the juvenile’s hiding potential (Baeza-Rojano et al., 2013a) or the use of devices or procedures that allow the separation of juveniles when they are released from the female’s brood pouch (Bloor, 2010).

## Conclusions

Amphipods can be interesting alternatives to traditional live foods such as *Artemia*. The present study assessed for the first time the aquaculture potential of two cosmopolitan marine gammarids that naturally proliferate in the wild and in aquaculture facilities. *P. hawaiensis,* unlike *E. pectenicrus,* performed well under laboratory conditions, showed a large total length (9.3 ± 1.3 mm) and body mass (14.4 ± 6.2 mg wet weight), with the ability to grow almost equally well using different cheap diets and without negative effects on fecundity, highlighting their excellent aquaculture potential. However, *P. hawaiensis* showed quite long generation times (50.9 ± 5.8 days) and relatively low fecundity levels (12.8 ± 5.7 embryos per female). As a result, production numbers were not impressive (0.36 ± 0.08 juveniles per couple per day) but may be sufficient to sustain the aquaculture of species that require low food quantities such as seahorses and other highly-priced marine ornamental organisms, where food quality is more important than food quantity. Future studies should better optimize the *P. hawaiensis* culture, assess their nutritional composition and to explore medium- and large-scale production. By contrast, the culture of *E. pectenicrus* was not successful under the present culture conditions, since adult individuals were adequately maintained in captivity but failed to reproduce. More information on the feeding habits and the reproductive biology of *E. pectenicrus* is needed to better assess its aquaculture potential. Results herein also highlighted the importance of sex ratio and cannibalism in maximizing amphipod production. These results contributed to the advance of the marine amphipod aquaculture.

##  Supplemental Information

10.7717/peerj.10840/supp-1 Supplemental Information 1 Numerical data on gammarid amphipod several response variables used to construct the graphics, tables and statistic analysis Data on the different sections of the manuscript: biometry, gross energy content, generation time, in situ sex ratio, fecundity, pilot culture, effect sex ratio on fecundity, cannibalism, effect of diet on fecundity, effect of diet on growth.

10.7717/peerj.10840/supp-2 Supplemental Information 2 Statistical analyses performed and summary results One-tailed t tests, one-way ANOVAs and Regression analysis statistical results on several response variable comparisons used on gammarid amphipod zootechnical culture protocols.

## Additional Information and Declarations

### Data Availability

The following information was supplied regarding data availability: Raw data on the several variables used to prepare the different contribution graphics sections, plus a second file with the statistical analysis detailed metrics are available at Zenodo: Vargas-Abúndez, Jorge Arturo, López-Vázquez, Humberto Ivan, Mascaró, Maite, Martínez-Moreno, Gemma Leticia, & Simões, Nuno. (2020). Marine amphipods as a new live prey for ornamental aquaculture: exploring the potential of Parhyale hawaiensis and Elasmopus pectenicrus (Version 1) [Data set]. Zenodo. http://doi.org/10.5281/zenodo.4146056.
