# Supplementary material for: Marine amphipods as a new live prey for ornamental aquaculture: exploring the potential of Parhyale hawaiensis and Elasmopus pectenicrus
Source: PeerJ. 2021 Feb 10;9:e10840. doi: 10.7717/peerj.10840 (PMC7881717; doi:10.7717/peerj.10840)
Supplement: Supplemental Information 2 — One-tailed t tests, one-way ANOVAs and Regression analysis statistical results on several response variable comparisons used on gammarid amphipod zootechnical culture protocols. [file peerj-09-10840-s002.docx]

| Table 1: Statistical analyses performed and summary results | | | | | |
| --- | --- | --- | --- | --- | --- |
| Analysis | Comparison | Statistic | Probability | Degrees of freedom | Transform |
| One-tailed t test | Total length by species | t = 7.9 | < 0.0001 | 195 | - |
|  | Total length by sex (*P. hawaiensis*) | t = 3.6 | = 0.0002 | 98 | - |
|  | Total length by sex (*E. pectenicrus*) | t = 3.6 | = 0.0003 | 95 | - |
|  | Wet weight by species | t = 7.9 | < 0.0001 | 194 | - |
|  | Dry weight by species | t = 8.7 | < 0.0001 | 194 | - |
|  | Wet weight by sex (*P. hawaiensis*) | t = 5.0 | < 0.0001 | 98 | - |
|  | Wet weight by sex (*E. pectenicrus*) | t = 5.2 | < 0.0001 | 95 | - |
|  | Size of first precopula by sex (*P. hawaiensis*) | t = 11.28 | < 0.0001 | 208 | - |
|  | Fecundity by species | t = 7.1 | < 0.0001 | 143 | - |
|  | Cannibalism by sex (*P. hawaiensis*) | t = 2.2 | = 0.0298 | 8 | - |
| One-way ANOVA | Gross energy content | F = 85.68 | < 0.0001 | 2, 12 | - |
|  | Effect of sex ratio on fecundity (*P. hawaiensis*) | F = 14.4 | = 0.0016 | 2, 9 | Y=Ln(Y) |
|  | Effect of sex ratio on fecundity (*E. pectenicrus*) | F = 2.6 | = 0.1286 | 2, 9 | - |
|  | Effect of diet on fecundity (*P. hawaiensis*) | F = 0.6 | = 0.5572 | 2, 12 | - |
|  | Effect of diet on total length at day 30 (*P. hawaiensis*) | F = 3.3 | = 0.0461 | 2, 51 | - |
| Regression analysis | ln Wet weight vs ln Total length (*P. hawaiensis*) | F = 656.8 | < 0.0001 | 1, 97 | X=Ln(X); Y=Ln(Y) |
|  | ln Wet weight vs ln Total length (*E. pectenicrus*) | F = 552.7 | < 0.0001 | 1, 95 | X=Ln(X); Y=Ln(Y) |
|  | Wet weight vs dry weight (*P. hawaiensis*) | F = 1988 | < 0.0001 | 1, 97 |  |
|  | Wet weight vs dry weight (*E. pectenicrus*) | F = 3795 | < 0.0001 | 1,95 |  |
|  | Fecundity vs total length (*P. hawaiensis*) | F = 40.3 | < 0.0001 | 1, 91 |  |
|  | Fecundity vs total length (*E. pectenicrus*) | F = 3.7 | = 0.0611 | 1, 50 |  |
